# Ultrasound and Microbubble-targeted Delivery of a microRNA Inhibitor to the Heart Suppresses Cardiac Hypertrophy and Preserves Cardiac Function

**DOI:** 10.7150/thno.34895

**Published:** 2019-09-21

**Authors:** Jonathan A. Kopechek, Charles F. McTiernan, Xucai Chen, Jianhui Zhu, Maureen Mburu, Rafey Feroze, Daniel A. Whitehurst, Linda Lavery, Jissy Cyriac, Flordeliza S. Villanueva

**Affiliations:** 1 Center for Ultrasound Molecular Imaging and Therapeutics, Heart and Vascular Institute, Pittsburgh Heart, Lung, Blood and Vascular Medicine Institute, University of Pittsburgh, Pittsburgh, PA, USA; 2 Dept. of Bioengineering, University of Louisville, Louisville, KY, USA

**Keywords:** Cardiac hypertrophy, Ultrasound targeted microbubble cavitation, miRNA delivery

## Abstract

MicroRNAs (miRs) are dysregulated in pathological left ventricular hypertrophy. AntimiR inhibition of miR-23a suppressed hypertension-induced cardiac hypertrophy in preclinical models, but clinical translation is limited by a lack of cardiac-targeted delivery systems. Ultrasound-targeted microbubble cavitation (UTMC) utilizes microbubbles as nucleic acid carriers to target delivery of molecular therapeutics to the heart. The objective of this study was to evaluate the efficacy of UTMC targeted delivery of antimiR-23a to the hearts of mice for suppression of hypertension-induced cardiac hypertrophy.

**Methods:** Cationic lipid microbubbles were loaded with 300 pmol negative control antimiR (NC) or antimiR-23a. Mice received continuous phenylephrine infusion via implanted osmotic minipumps, then UTMC treatments with intravenously injected antimiR-loaded microbubbles 0, 3, and 7 days later. At 2 weeks, hearts were harvested and miR-23a levels were measured. Left ventricular (LV) mass and function were assessed with echocardiography.

**Results:** UTMC treatment with antimiR-23a decreased cardiac miR-23a levels by 41 ± 8% compared to UTMC + antimiR-NC controls (*p* < 0.01). Furthermore, LV mass after 1 week of phenylephrine treatment was 17 ± 10% lower following UTMC + antimiR-23a treatment compared to UTMC + antimiR-NC controls (*p* = 0.02). At 2 weeks, fractional shortening was 23% higher in the UTMC + antimiR-23a mice compared to UTMC + antimiR-NC controls (*p* < 0.01).

**Conclusions:** UTMC is an effective technique for targeted functional delivery of antimiRs to the heart causing suppression of cardiac hypertrophy and preservation of systolic function. This approach could represent a revolutionary therapy for patients suffering from pathological cardiac hypertrophy and other cardiovascular conditions.

## Introduction

Pathological left ventricular hypertrophy (LVH), such as occurs in hypertensive heart disease, is associated with an increased risk of heart failure and other cardiovascular complications 1-8. Pathological LVH is characterized by significant changes in gene expression which lead to ventricular remodeling 9. The mechanisms by which pathological LVH confers increased cardiovascular risks are complex, and may include increased susceptibility to myocardial ischemia due to reduced capillary density, impaired flow reserve, endocardial capillary compression, and/or electrical remodeling 10-13. Compared to the development or persistence of LVH 14, 15, prevention or regression of LVH is clinically associated with improved LV systolic performance and reduced risk of cardiovascular events (myocardial infarction, stroke, sudden death). Anti-hypertensive medications have some effect on pathological LVH: a meta-analysis found that angiotensin converting enzyme inhibitors and angiotensin receptor blockers reduced LV mass by 10% and 13%, respectively 16, 17. However, these medications can cause side effects 18, 19 and are not uniformly effective, underscoring the need for new therapies to mitigate the development of LVH.

Dysregulation of microRNA (miRNA) activity has been shown to play an important role in the development of cardiovascular conditions such as LVH, and therapeutic modulation of miRNA levels in the heart has generated interest for treatment of various cardiovascular diseases 20, 21. miRNAs are short (~22 nucleotides), non-coding, endogenous RNAs that regulate multiple genes and target multiple signaling pathways by binding target mRNA sequences to inhibit translation or induce mRNA degradation 21. Preclinical studies have demonstrated that levels of miRNAs are dynamically altered in the heart under pathological conditions, including hypertrophy, and studies of failing human hearts detected significant changes in levels of specific miRNAs compared to normal hearts 22-24. One particular miRNA, miR-23a, was found to be significantly upregulated in human hearts with ischemic cardiomyopathy, dilated cardiomyopathy, or aortic stenosis, and among the earliest miRNAs to be upregulated following aortic banding in rat hearts 23, 25. Preclinical studies found that miR-23a promotes hypertrophy by upregulating the calcineurin/NFAT pathway and through inhibition of ubiquitin proteolysis via factors such as MuRF1, regulation of transcription factors such as Foxo3a, and downregulation of anti-oxidant enzymes such as MnSOD 26, 27. Inhibition of miR-23a using an antimiR suppressed cardiac hypertrophy in mice with pressure overload or hypertension-induced hypertrophy 26, 27. AntimiRs have also shown promise for treatment of other diseases and are in clinical trials for treatment of hepatitis C 28. However, systemic administration of miRNA therapeutics such as antimiRs requires high doses and can adversely affect miRNA levels in off-target organs 21, 29, 30.

To address limitations with systemic antimiR delivery, we have developed a novel approach to locally deliver RNA therapeutics with ultrasound targeted microbubble cavitation (UTMC). This platform utilizes a multi-purpose gas-filled microsphere (microbubble) which carries the therapeutic on the shell, serves as an ultrasound imaging contrast agent, and can facilitate trans-membrane transport of drugs and genes in response to ultrasound 31-33. As intravenously injected microbubbles transit though the microcirculation of the target tissue, ultrasound directed at the target tissue causes microbubble destruction or cavitation and payload release, and enhances vascular permeability and cell membrane permeability only at the target site 34-36. This approach allows for repeated administration of targeted molecular therapeutics without the use of viral vectors. Previous studies by us and others have demonstrated the efficacy of UTMC for delivery of transcription factors, siRNA, and plasmids to the heart and other tissues 37-40.

We previously loaded luciferase siRNA onto cationic lipid-coated microbubbles and demonstrated cardiac delivery with UTMC in mice with cardiac-restricted luciferase expression 40. In addition, other studies have explored UTMC delivery of miRNA mimics to cardiomyocytes *in vitro* and accumulation of fluorescent antimiRs in mouse hearts following UTMC 41-43. However, the functional effects of therapeutic antimiR delivery via UTMC have not been previously described in a preclinical model of cardiac disease. Therefore, we hypothesized that UTMC could target delivery of antimiR-23a to cardiomyocytes, knock down miR-23a levels, suppress cardiac hypertrophy, and preserve cardiac function. Studies were initially performed in cultured murine cardiomyocytes *in vitro,* followed by *in vivo* experiments in a murine model of hypertension-induced LVH.

## Methods

### Experimental animals

All animal procedures conformed to NIH guidelines for the care and use of laboratory animals and were approved by the Institutional Animal Care and Use Committee at the University of Pittsburgh. A total of 22 male C57/BL6 mice were used for this study. For all surgical procedures, mice were anesthetized using 2-3% inhaled isoflurane and the depth of anesthesia was monitored by toe pinch response. All mice were euthanized by resection of the heart and decapitation under deep isoflurane anesthesia (5%).

### Isolation of cardiomyocytes

**Figure 1:**
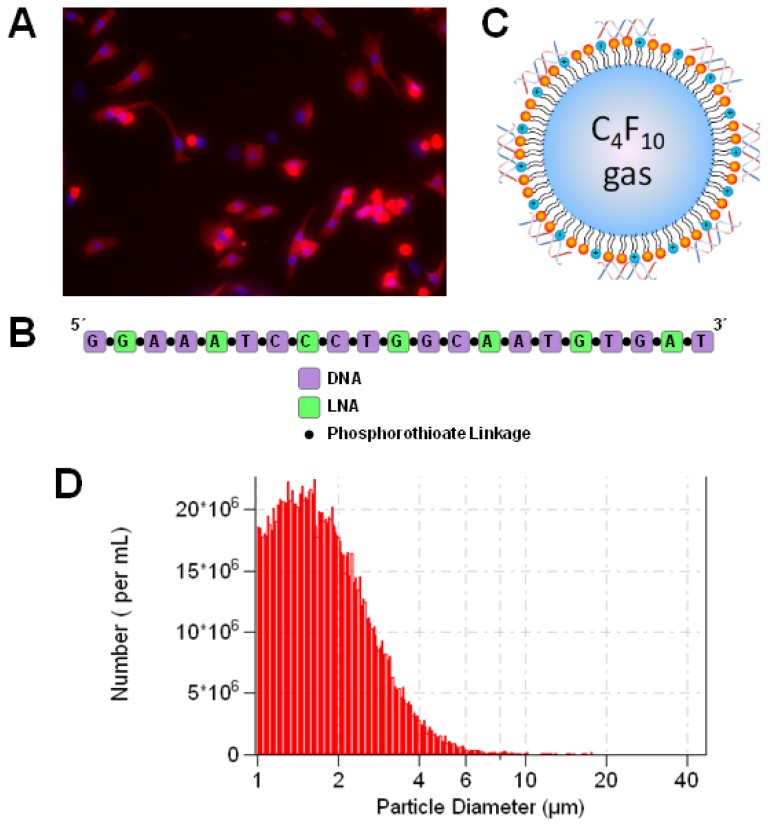
** Microbubbles loaded with antimiR-23a for ultrasound-mediated therapeutic delivery.** (A) Fluorescence microscopy image of myosin-stained cells indicating cardiomyocyte purity of >80%. Nuclei were stained with Hoechst (blue). (B) Sequence and structure of LNA-modified antimiR-23a. (C) Schematic rendering of antimiR-loaded cationic lipid-coated microbubbles. (D) Size distribution of microbubbles loaded with antimiR-23a, as measured by Coulter counter.

Neonatal ventricular cardiomyocytes were isolated from the hearts of 1-2 day old Sprague-Dawley rats following manufacturer's directions with the Neonatal Cardiomyocyte Isolation System (Worthington Biochemical, Lakewood, NJ, USA). Cardiomyocytes were cultured on plates coated with 10 µg/cm^2^ rat tail collagen (Sigma-Aldrich, St. Louis, MO, USA). All cell studies were initiated 48 h after isolation. Myosin staining of the cells indicated that the cardiomyocyte purity was above 80% (Figure 1A).

### AntimiR-loaded microbubble preparation and characterization

LNA-modified antimiRs were obtained from Exiqon (Copenhagen, Denmark). The sequence and structure are illustrated in Figure 1B. The sequence of “antimiR-23a” (with LNA-modified bases underlined) was GGAAATCCCTGGCAATGTGAT. The sequence of the negative control antimiR, “antimiR-NC”, was CAGTACTTTTGTGTAGTACAA.

Cationic microbubbles were synthesized as previously described 39, 40. Lipids were obtained from Avanti Polar Lipids (Alabaster, AL, USA) and polyethylene glycol-40 (PEG-40) stearate was obtained from Sigma-Aldrich. Lipids and PEG-40 were dissolved in chloroform at a molar ratio of 100:43:1:4.5 of 1,2-distearoyl-sn-glycero-3-phosphocholine (DSPE), 1,2-distearoyl-sn-glycero-3-ethylphosphocholine (DSEPC), 1,2-distearoyl-sn-glycero-3-phosphoglycerol (DSPG), and PEG-40. The solution was dried with argon and resuspended at 1 mg/mL in phosphate buffered saline (PBS) containing 1 mM EDTA, followed by sonication (Misonix, Farmingdale, NY, USA) to disperse the lipids. The lipid solution was diluted 1:4 in PBS (with 1 mM EDTA) to a volume of 800 µL, and 10 μg of antimiR-23a or antimiR-NC was added. The solution was placed in a sealed glass vial and the head space was filled with perfluorobutane gas (FluoroMed, Round Rock, TX, USA), followed by amalgamation (VialMix, Bristol Myers-Squibb, New York, NY, USA) to form lipid encapsulated perfluorobutane gas-filled microbubbles loaded with antimiRs via charge-charge interactions with the cationic lipid membrane (Figure 1C). AntimiR-loaded microbubbles were washed in PBS three times with centrifugation at 50g to remove unbound antimiR and lipids, and resuspended in a final volume of 0.5 mL. The resulting antimiR-loaded microbubble suspension contained approximately 2×10^9^ microbubbles/mL with a mean diameter of 2.0±0.1 μm (Figure 1D) as measured by a Coulter Multisizer 3 (Beckman Coulter, Miami, FL, USA). Loading of antimiR-23a on microbubbles was assessed quantitatively and qualitatively using methylene blue electrophoresis. Methylene blue staining was performed as previously described 44. Following washing, 100 µL microbubbles were briefly treated in a sonicating water bath to disrupt the microbubble membrane, and heparin (200 U/mL) was added to dissociate antimiR from the lipid microbubble components prior to running the electrophoresis gel. A representative gel is shown in Online Figure S1 indicating that 40% of the antimiR added to the formulation could be loaded on to the microbubbles.

### UTMC treatment with antimiR *in vitro*

**Figure 2:**
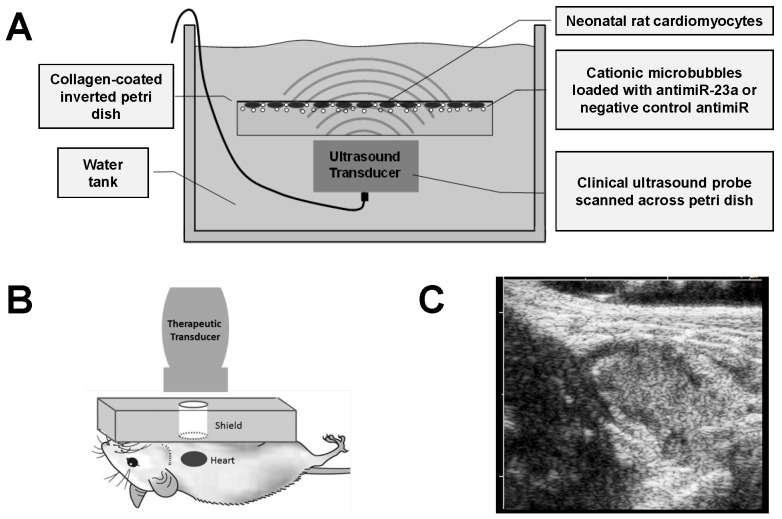
** Experimental setup for *in vitro* and *in vivo* ultrasound studies.** (A) Schematic of experimental setup for *in vitro* studies of UTMC antimiR delivery to cardiomyocytes. (B) Schematic of experimental setup for *in vivo* studies of UTMC antimiR delivery to mouse hearts. AntimiR-loaded microbubbles were injected through the jugular vein and insonified in the heart with pulses from a clinical ultrasound imaging system. (C) Representative B-mode ultrasound image of nucleic acid-loaded microbubbles in mouse heart (acquired with Siemens Acuson ultrasound system).

Studies were performed to evaluate UTMC delivery of antimiR to cardiomyocytes *in vitro* as illustrated in Figure 2A. Cardiomyocytes were plated on collagen-coated (10 µg/cm^2^) 35 mm petri dishes for 48 h. AntimiR-loaded microbubbles (5 million or 10 million microbubbles with 3.5 or 7.0 pmol of antimiR, respectively) were added to cardiomyocyte cultures in DMEM/Ham's F12 media (Corning Inc., Corning, NY, USA) with 2% fetal bovine serum (Atlanta Biologicals, Flowery Branch, GA, USA). The plates were sealed with parafilm, inverted, and incubated for 10 minutes to allow microbubbles to float to the top where cells were located. The inverted dishes were placed in a 37°C water bath and insonified using a Philips S-3 probe on a clinical ultrasound imaging system (Sonos 7500, Philips Healthcare, Bothell, WA, USA) in ultraharmonic mode (1.3 MHz, MI on-screen = 1.6, 4 frames/burst, 4 bursts/second). The MI of 1.6 was used for *in vitro* experiments to compensate for attenuation of ultrasound waves through the plastic petri dish, which reduced the pressure experienced by the plated cells. The transducer was scanned across the plate twice using the motorized controller on the Therapy Imaging Probe System (TIPS, Philips Healthcare) to expose the entire sample to ultrasound energy. Immediately following UTMC, the media was replaced with fresh media and samples were returned to the cell incubator. After UTMC treatment, phenylephrine (100 µM) was added to cardiomyocyte samples for varying durations (6-48 h) to induce hypertrophy.

### UTMC treatment *in vivo*

Studies were performed to evaluate UTMC delivery of antimiR to mouse hearts *in vivo* (Figure 2B). A venous cannula for microbubble infusion was surgically placed in the jugular vein of anesthetized mice. An osmotic minipump (model 2002, Alzet Corporation, Santa Clara, CA, USA) was loaded with phenylephrine (100 mg/kg/d) and placed subcutaneously on day 0. UTMC treatments were performed on days 0, 3, and 7, following minipump placement. This dosing schedule was based on our prior work with UTMC-mediated delivery of STAT3 decoy to tumors 39. A subset of five control animals did not receive phenylephrine infusion or UTMC treatment.

The clinical transducer used for this study transmits an ultrasound beam appropriately scaled in size for human echocardiography, but entirely covers both lung fields of the mouse. We thus considered the potential for air within the lungs to cause strong reflection, leading to constructive interference of the transmitted wave, resulting in regions of high pressure within the lungs and the potential for lung tissue damage. The lungs were therefore shielded from ultrasound by using acoustic absorbing material (polyurethane rubber, 1 cm thickness) with a circular window (10 mm diameter) placed on the chest overlying the heart, as previously described 40. Correct alignment of the window over the heart was confirmed using B-mode imaging with a 15L8 probe on a Siemens clinical ultrasound imaging system (14 MHz, Sequoia 512, Siemens Healthcare, Erlangen, Germany). Following alignment, UTMC treatment was applied using a clinical ultrasound imaging system (S3 probe, Sonos 7500, Philips Healthcare) in ultraharmonic mode (1.3 MHz, MI on-screen = 1.0, 4 consecutive frames/burst, 1 burst/second). The repetition rate of 1 burst/second was chosen empirically based on the time required for microbubbles to replenish the myocardium after each burst, as indicated by simultaneous contrast-enhanced echo imaging. Ultrasound pulses were not gated to the cardiac cycle due to the fact that each ultrasound burst spans an entire cardiac cycle in mice, who have heart rates greater than 500 bpm; gating the ultrasound pulses to the cardiac cycle would be possible in humans, whose heart rates are significantly less than that of mice.

AntimiR-loaded microbubbles (100 µL of microbubbles with 35 pmol of antimiRs) were continuously infused through the jugular cannula for 15 minutes at a rate of 0.4 mL/h during ultrasound treatment (Figure 2C). Ultrasound was applied for an additional 5 minutes post-infusion to insonify residual microbubbles in circulation (total ultrasound duration of 20 minutes).

To assess cardiac mass and function, echocardiography was performed at baseline, 1 week, and 2 weeks after phenylephrine pump implantation using a 30 MHz probe on a Vevo 2100 high-frequency ultrasound imaging system (VisualSonics, Toronto, CAN). Long-axis and short-axis B-mode and M-mode images were acquired on lightly anesthetized mice (heart rate > 400 bpm). Cardiac measurements were performed on short-axis M-mode images by an observer blinded to experimental condition using Vevo LAB software (VisualSonics) to determine LV mass, LV anterior and posterior wall thicknesses, LV internal diameter, and fractional shortening 45. Mice were euthanized after the 2 week echocardiograms were obtained. LV mass was calculated by the Vevo LAB software using the following equation:

LV mass = 0.8424 × [(IVSd + LVIDd + PWd)^3^ - LVIDd^3^],

where IVSd is the diastolic intraventricular septal thickness, LVIDd is the diastolic left ventricular internal diameter, and PWd is the diastolic posterior wall thickness.

### Analysis of cell area and molecular expression levels

For *in vitro* studies, the cross-sectional area of cardiomyocytes was measured by a blinded observer using brightfield microscopy images acquired at 10× magnification. Cells were harvested in TRIzol (Thermo Fisher Scientific, Waltham, MA, USA) for analysis of miRNA expression. Total RNA was isolated using the miRNeasy kit (Qiagen, Hilden, Germany). Levels of mature miR-23a were assessed using the miScript assay (Qiagen) and normalized by U6 levels.

For *in vivo* studies, harvested mouse hearts were placed in ice-cold cardioplegia solution and sectioned. Heart weights were measured and transverse sections were fixed in 4% formalin and paraffin embedded for staining with FITC-wheat germ agglutinin. Cardiomyocyte cross-sectional areas were measured manually by a blinded observer using fluorescence microscopy images acquired at 20× magnification. For analysis of miRNA levels, tissue sections were homogenized in TRIzol and miRNA levels were quantified following the same procedure described for *in vitro* studies. Levels of hypertrophic mRNAs (ANP and MHY7) were assessed using RT-PCR.

### Statistical Analysis

Comparisons between two experimental groups were determined using a Student's *t*-test, with statistical significance defined as *p* < 0.05 (two-tailed). Comparisons among more than two experimental groups were performed using analysis of variance (ANOVA), with significance defined as *p* < 0.05. Post-hoc comparisons were performed using Tukey's test. Bars represent mean ± SEM.

## Results

### Suppression of cardiomyocyte hypertrophy *in vitro* with UTMC and antimiR-23a

Studies were performed *in vitro* to evaluate the effect of ultrasound treatment with antimiR-loaded microbubbles on neonatal rat cardiomyocytes. To first assess whether UTMC, by itself, affected cell viability, trypan blue and MTT assays were performed following UTMC treatment with negative control (NC) antimiR-loaded microbubbles at a dose of either 5 million or 10 million microbubbles per sample (Online Figure S2). Cell viability was 88-98% as measured with trypan blue assay 30 minutes after UTMC or MTT assay 24 h after UTMC (normalized to no treatment control). Viability was retained at both microbubble doses and there were no statistically significant differences between each dose.

**Figure 3:**
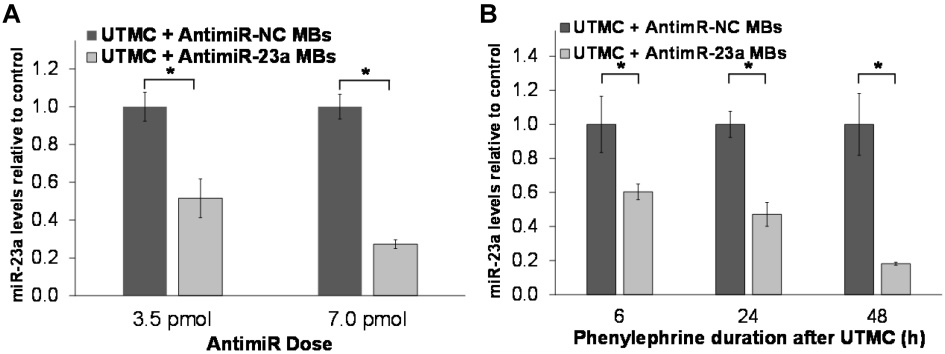
** Ultrasound delivery of antimiR-23a reduces miR-23a in cardiomyocytes *in vitro*.** UTMC-targeted delivery of antimiR-23a reduced miR-23a levels relative to each negative control condition at various (A) doses (after 24 h phenylephrine stimulation) and (B) duration of phenylephrine exposure (*n*=5-6/group at 3.5 pmol, *n*=3-6/group at 7.0 pmol, *n*=6/group at 6 h, *n*=9/group at 24 h, *n*=3/group at 48 h); *p<0.05.

The effect of a single UTMC treatment on miR-23a levels in phenylephrine-treated cardiomyocytes was assessed *in vitro* using RT-PCR. As shown in Figure 3A, miR-23a levels after 24h phenylephrine stimulation were significantly lower following a single UTMC + antimiR-23a microbubble treatment at two different doses compared to the corresponding negative control conditions (UTMC + antimiR-NC microbubbles). Furthermore, it was found that miR-23a levels continued to be less, even after 48 h of phenylephrine exposure, in samples treated with UTMC + antimiR-23a microbubbles (3.5 pmol antimiR) compared to the negative control condition (UTMC + 3.5 pmol antimiR-NC microbubbles) (Figure 3B). In comparison, miR-23a levels in cardiomyocytes strongly tended to increase, up to several-fold (*p* = 0.13), within 6 h of phenylephrine exposure and remained elevated for up to 72 h (Online Figure S3A). In addition, treatment with antimiR-23a alone (without UTMC) had no significant effect on miR-23a levels in phenylephrine-treated cardiomyocytes (Online Figure S3B), demonstrating that ultrasound and microbubbles were necessary to effectively deliver antimiR-23a to cardiomyocytes and knock down miR-23a levels at the doses tested in this *in vitro* study.

**Figure 4:**
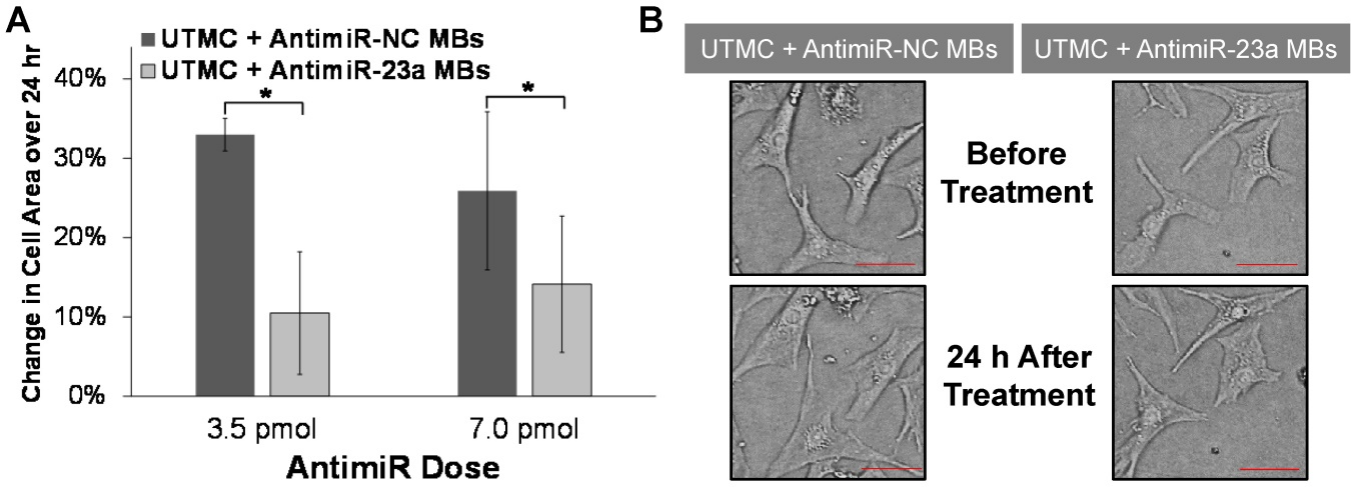
** Ultrasound delivery of antimiR-23a reduces cardiomyocyte hypertrophy *in vitro*.** (A) UTMC-targeted delivery of antimiR-23a at two different antimiR doses suppressed cardiomyocyte hypertrophy after 24 h of phenylephrine exposure compared to antimiR-NC delivery at the same doses (*n*=3-4/group); *p<0.05. (B) Representative microscopy images of cardiomyocytes before and after phenylephrine stimulation and UTMC treatment with 3.5 pmol of antimiR-23a or antimiR-NC (scale bar = 10 µm).

Histologic studies demonstrated that UTMC targeted delivery of antimiR-23a suppressed phenylephrine-induced cardiomyocyte hypertrophy *in vitro*. As shown in Figure 4, after 24 h of phenylephrine exposure, increase in cell area was significantly less after UTMC + antimiR-23a microbubble treatment compared to UTMC + antimiR-NC microbubble treatment (at two different antimiR doses). These results indicate that the reduced cardiomyocyte levels of miR-23a resulting from UTMC delivery of antimiR-23a *in vitro* caused the biologically predicted consequences of miR-23a antagonism.

### Suppression of phenylephrine-induced cardiac hypertrophy *in vivo* with UTMC and antimiR-23a

**Figure 5:**
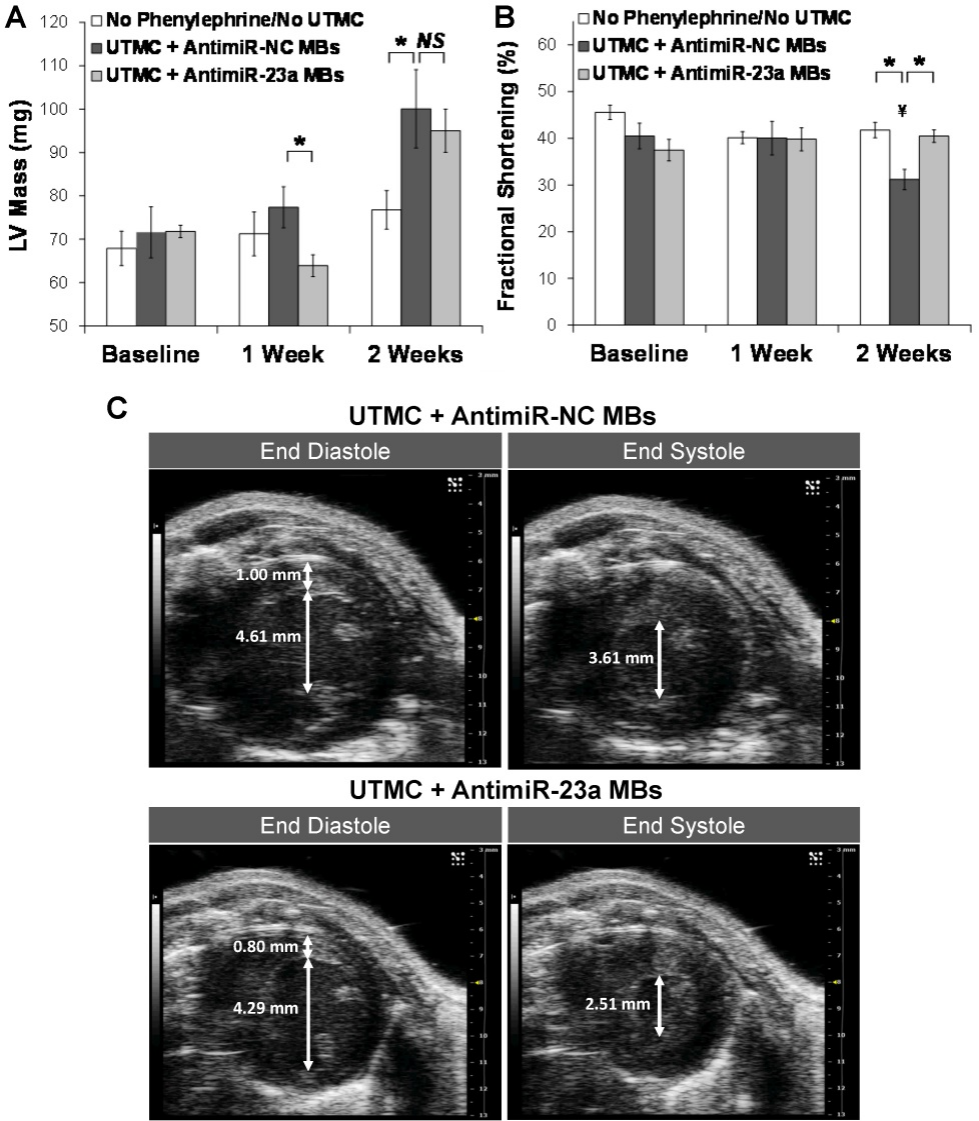
**Ultrasound delivery of antimiR-23a reduces cardiac hypertrophy and improves cardiac function *in vivo*.** Echocardiographic analysis indicating suppression of phenylephrine-induced left ventricular hypertrophy and preservation of systolic function with UTMC targeted delivery of antimiR-23a compared to UTMC delivery of antimiR-NC. The “no phenylephrine/no UTMC” group received neither phenylephrine nor ultrasound. Compared to UTMC + antimiR-NC microbubbles, UTMC + antimiR-23a microbubble treatment (A) blunted development of LV hypertrophy at 1 week (*p=0.02), and (B) preserved fractional shortening at 2 weeks (*p<0.01, ¥ *p*<0.02 vs baseline, *n*=5-9 animals/group). (C) Representative end diastolic (left) and end systolic (right) B-mode frames at 2 weeks in two mice, showing preservation of left ventricular systolic function with UTMC + antimiR-23a microbubbles (lower panels) compared to control (upper panels). Scales (right side of each image) are the same for all frames. In these images the measured LV wall thickness at end-diastole was 0.80 mm and 1.00 mm with UTMC + antimiR-23a MB treatment and control treatment, respectively. The internal diameter at end-diastole was 4.29 mm and 4.61 mm with UTMC + antimiR-23a MB treatment and control treatment, respectively. The internal diameter at end-systole was 2.51 mm and 3.61 mm with UTMC + antimiR-23a MB treatment and control treatment, respectively. MB = microbubbles.

Studies were performed *in vivo* to assess the effect of ultrasound treatment with antimiR-loaded microbubbles on phenylephrine-induced cardiac hypertrophy and cardiac function in mice. As shown in Figure 5A, the two UTMC-treated groups of mice developed progressive left ventricular hypertrophy over the course of two weeks of phenylephrine infusion, which was blunted in mice treated with UTMC + antimiR-23a microbubbles, with LV mass after 1 week of phenylephrine treatment being significantly less in this group compared to that in the UTMC + antimiR-NC microbubble group (*p* = 0.02). At 2 weeks (1 week after the final UTMC treatment) LV mass remained numerically lower in the mice treated with UTMC + antimR-23a microbubbles compared to mice treated with UTMC + antimiR-NC microbubbles, but the difference was no longer statistically significant. Figure 5B shows that fractional shortening at baseline was similar among the mice treated with no phenylephrine/no UTMC, and phenylephrine-treated mice receiving UTMC + antimiR23a or + antimiR-NC microbubbles. By 2 weeks, fractional shortening in the UTMC + antimiR-23a microbubble group was unchanged from baseline, whereas fractional shortening in the UTMC + antimiR-NC microbubble group had decreased from baseline by 23% (*p* < 0.02), such that it was significantly less than that in the UTMC + antimiR-23a microbubble group (*p* < 0.01). Also, compared to the no phenylephrine/no UTMC control group at 2 weeks, fractional shortening was no different in the UTMC + antimiR-23a microbubble group, but 25% less in the UTMC + antimiR-NC microbubble group at 2 weeks (*p* < 0.01), LV anterior and posterior wall thickness tended to be lower at 1 week in phenylephrine-treated mice receiving UTMC + antimiR-23a microbubbles compared to UTMC + antimiR-NC microbubbles, although the differences did not achieve statistical significance (Online Figure S4A and S4B, respectively). As shown in Online Figure S4C, at 2 weeks after phenylephrine minipump placement, LV end systolic diameter of UTMC + antimir-23a microbubble-treated mice was unchanged from baseline and no different from that in mice receiving no phenylephrine/no UTMC. In contrast -- and in line with the fractional shortening data (Figure 5B) -- by 2 weeks, LV end systolic diameter had increased in the mice receiving UTMC + antimiR-NC microbubbles (*p* = 0.03 vs. baseline) such that it was higher than that in mice treated with UTMC + antimir-23 (*p* = 0.06; Online Figure S4C). Figure 5C shows representative left ventricular end diastolic and end systolic frames from two mice at 2 weeks of phenylephrine infusion after UTMC treatment with antimiR-23a microbubbles (lower panels) or antimiR-NC microbubbles (upper panels), demonstrating preservation of systolic function in the antimiR-23a-treated mouse compared to the antimiR-NC control.

**Figure 6:**
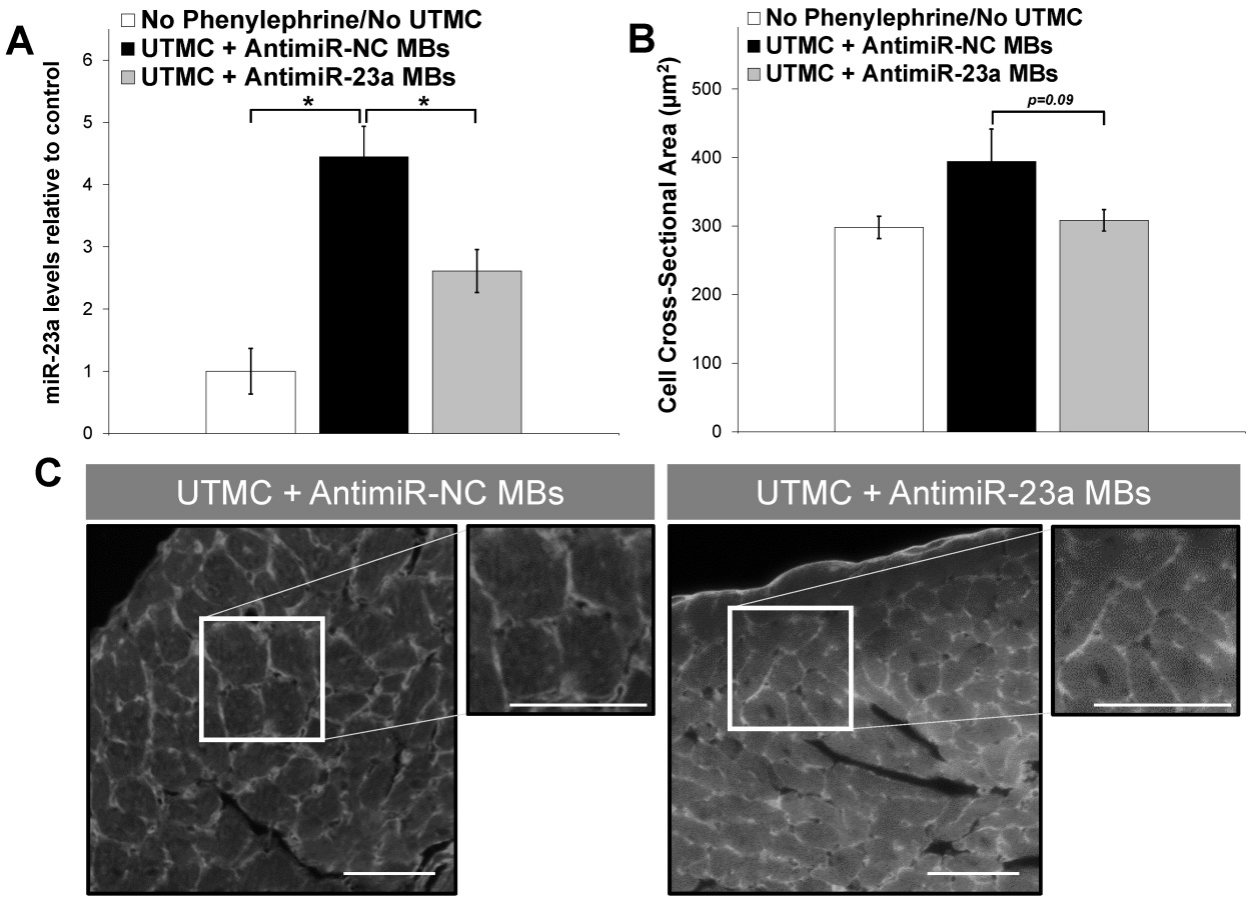
** Ultrasound delivery of antimiR-23a reduces miR-23a levels and suppresses cardiomyocyte hypertrophy *in vivo*.** (A) UTMC delivery of antimiR-23a suppressed phenylephrine-induced elevation of miR-23a levels in mouse hearts compared to UTMC delivery of antimiR-NC (*p<0.01, *n*=5-9/group). (B) Cardiomyocyte size strongly tended to be less following UTMC delivery of antimiR-23a compared to UTMC delivery of antimiR-NC, (*p*=0.09, *n*=5-9/group). (C) Representative fluorescence microscopy images of wheat germ agglutinin-stained left ventricular sections, demonstrating that UTMC treatment with antimiR-23a microbubbles suppressed phenylephrine-induced cell growth. Scale bar represents 40 µm.

Mouse hearts were harvested at the end of the 2-week protocol and processed to analyze miR-23a levels, mRNA levels of hypertrophy markers, and to determine cardiomyocyte cross-sectional area in wheat germ agglutinin-stained sections. As shown in Figure 6A, there was a 4.5-fold increase in miR-23a levels in phenylephrine-treated mice receiving UTMC + antimiR-NC microbubble (*p*<0.001 vs. untreated no-phenylephrine group); miR-23a levels also increased in UTMC + antimiR-23a microbubble treated mice, but to a lesser extent (2.6-fold compared to the untreated no-phenylephrine group), and were significantly less than that in the UTMC + antimiR-NC microbubble treated mice (*p* < 0.01 vs. UTMC + antimiR-NC group). Furthermore, cardiomyocyte size following UTMC delivery of antimiR-23a tended to be less than that following UTMC + antimiR-NC microbubble treatment (*p* = 0.09, Figure 6B). Cardiac levels of hypertrophy markers ANP and MYH7 mRNA were less at 2 weeks in phenylephrine-treated mice receiving UTMC + antimiR-23a microbubble treatments compared to treatment with UTMC + antimiR-NC microbubbles (Online Figure S5) although the differences did not reach statistical significance. There was no difference between groups in post-mortem gross heart weight (Online Figure S6).

## Discussion

This study indicates that ultrasound targeted microbubble cavitation is an effective non-invasive approach for targeted delivery of antimiRs to the heart. *In vitro*, UTMC delivery of antimiR-23a to phenylephrine-treated cardiomyocytes blunted the rise in miR-23a levels and progressive cardiomyocyte hypertrophy that were seen in negative controls. Furthermore, in phenylephrine-treated mice, cardiac-directed UTMC delivery of antimiR-23a attenuated the rise in miR-23a levels, mitigated cardiac hypertrophy, and preserved cardiac function compared to ultrasound-targeted delivery of a negative control antimiR. We previously demonstrated functional cardiac delivery of siRNA against luciferase reporter using UTMC 40. Other investigators have previously explored UTMC delivery of antimiR to the heart in acute studies 42, 43. However, to our knowledge this is the first study to demonstrate suppression of a pathological cardiac condition (*i.e.* cardiac hypertrophy) following cardiac-targeted UTMC delivery of antimiRs.

Previous studies have demonstrated the anti-hypertrophic effect of antimiR-23a in mice receiving adrenergic agonists or transaortic constriction 26, 27. However, in the prior studies, antimiRs were administered systemically and therefore could potentially induce unwanted side effects in off-target tissues. For example, reduced levels of miR-23a is associated with atrophy of skeletal muscle 46-48. In addition, high doses (25-30 mg/kg/d for 7-14 days) were used in order achieve therapeutic effects in the heart. In our study, a dose of only 0.1 mg/kg was administered on 3 treatment days over 1 week. This suggests that UTMC can significantly reduce the antimiR dose needed for therapeutic efficacy (by a factor of over 200-fold). Only one dose was tested in this study but future experiments will compare the efficacy of other doses in order to determine whether this treatment approach is effective at doses even lower than the 0.1 mg/kg used in this study.

UTMC treatments with antimiR were initiated at the beginning of phenylephrine infusion, similar to other studies of miRNA therapy 26, 27. Therefore, these preclinical studies have not addressed questions of whether treatment can cause regression of established cardiac hypertrophy. The results of the present study demonstrate proof-of-principle that UTMC is an effective strategy for functional delivery of antimiRs to the heart, providing groundwork for future studies aimed at determining whether this treatment strategy can attenuate or regress hypertrophy once it has already developed.

A limitation of our study is that the duration of antimiR activity resulting from UTMC was not fully defined. UTMC treatments were performed at days 0, 3, and 7 following initiation of phenylephrine infusion, and the study was terminated at day 14, one full week after the final UTMC treatment, with phenylephrine infusion ongoing throughout. The echocardiography results suggest that the functional effect of antimiR-23a delivery decreases over time: whereas UTMC-mediated delivery of antimiR-23a loaded microbubbles blunted phenylephrine-induced LVH at 1 week relative to controls, this difference was not sustained, and by 2 weeks into the protocol (1 week following the last UTMC treatment), LV mass was comparable between the UTMC + antimiR-NC-treated mice and UTMC + antimiR-23a-treated mice, in the presence of ongoing phenylephrine stimulation (Figure 5A). This finding was corroborated by the actual weights of the heart post-mortem (Online Figure S6).

Although myocardial miR-23a levels at 2 weeks in UTMC + antimiR-23a-treated mice were about half that in UTMC + antimiR-NC-treated mice, they were still twice that in non-phenylephrine treated (no UTMC) control mice (Figure 6), a relative difference which is similar to what we found *in vitro*. These levels of incomplete and relatively short term miR-23a inhibition were apparently insufficient to continue LVH suppression in the presence of ongoing phenylephrine infusion. Concordantly, this attenuation of therapeutic advantage as time elapsed after the last UTMC treatment was paralleled by the finding that levels of hypertrophy markers were not significantly reduced at 2 weeks in mice receiving UTMC + antimiR-23a microbubbles. Therefore, more treatments - past the 7 day timepoint used in our study - may have been be needed to maintain suppression of cardiac hypertrophy during ongoing phenylephrine exposure in this mouse model. Clinically, repeated treatments may also be needed to prevent progression of pathological LVH toward heart failure, but further preclinical studies would be needed to assess the effect of more frequent dosing schedules. In particular, our model was one of persistent hypertrophic stimulation with phenylephrine, ostensibly warranting ongoing antimiR-23a delivery to blunt the evolution of LVH. We would not anticipate the need for indefinite antimiR therapy in the clinical situation, where drivers of LVH (*e.g.* hypertension) would presumably also be treated. We do not expect that antimiR therapy would replace standard treatment strategies for management of LVH (i.e. oral drugs, exercise, weight loss) 49. Instead, we envision that this approach could be used to augment standard treatments and induce stronger suppression of LVH, particularly for patients who respond poorly to standard treatments. A meta-analysis found that angiotensin converting enzyme inhibitors and angiotensin receptor blockers reduced LV mass in patients by 10% and 13%, respectively 16, 17, which is not as strong as the level of suppression observed in this preclinical study after 1 week of UTMC treatment (18%). We expect that the combination of standard therapies with antimiR treatments could potentially further suppress cardiac hypertrophy and may improve patient outcomes.

Interestingly, despite the loss of protection against LVH by 1 week after the last treatment (2 weeks into the protocol), UTMC mediated antimiR-23a treatment appeared to protect against phenylephrine-induced LV systolic dysfunction. This may reflect a benefit from the treatment-induced delay in the onset of LVH. Whether the preservation in systolic function in the antimiR-23a treated group would have been sustained beyond 2 weeks is unknown. Our data suggest, however, that the mitigation of phenylephrine-induced LVH by UTMC mediated antimiR-23a delivery is associated with subsequent preservation of systolic function.

This study investigated UTMC delivery of a single antimiR sequence (antimiR-23a). However, this approach could be extended to load microbubbles with multiple antimiRs simultaneously for targeted delivery to the heart. In addition to antimiRs, miRNA mimics could also be loaded onto cationic microbubbles, potentially enabling simultaneous modulation of multiple miRNAs with this approach. Strategies to target UTMC delivery of antimiRs/miRNA mimics only to specific cell types (*i.e.* cardiomyocytes, fibroblasts, or endothelial cells) would also be important, as some miRNAs confer beneficial effects in certain cell types while having detrimental effects in others. In our study, it is unknown whether antimiR-23a delivery induced any effects in cardiac cells other than cardiomyocytes. We have initially focused on the established role of miR-23a in cardiomyocyte hypertrophy but future studies are needed to elucidate the effects of antimiR-23a therapy on other cardiac cell types such as fibroblasts, endothelial cells, monocytes, and macrophages.

This approach could be effective in restoring levels of multiple miRNAs to normal levels, which could improve therapeutic responses where multiple signaling pathways, as is usually the case, are involved in the pathologic response. However, the functions of many miRNAs have not yet been well characterized and even less is known about the effects of manipulating multiple miRNAs simultaneously. In addition, these aspects will need to be explored further in future preclinical studies, including with large animal models such as swine, before clinical translation in humans can occur.

UTMC has potential adverse bioeffects that can occur under certain conditions. Prior studies have shown that UTMC can cause petechiae or hemorrhage, particularly in the lungs, when very high microbubble doses or high acoustic pressures are administered 50. In our study, however, we used a clinical ultrasound imaging system which limits the acoustic pressures to levels that are considered safe for humans by regulatory agencies such as the FDA. Furthermore, in this study we used a microbubble dose which we previously tested in mice, with no damage or hemorrhage detected in cardiac tissues 40. To maximize the antimiR dose that could be delivered, we maximally loaded the microbubbles and used a total microbubble dose that was higher than that currently FDA-approved for human use (10 µL/kg for Definity, 51). We did not however, observe adverse effects in the mice. Future studies will explore lower microbubble doses to find the therapeutic window and to determine the minimal dose for effective antimiR therapy.

In summary, we have demonstrated that ultrasound targeted microbubble cavitation is an effective, non-invasive technique for targeted functional delivery of antimiRs to the heart. This study indicated for the first time that UTMC delivery of antimiRs can suppress pathological cardiac hypertrophy and preserve left ventricular systolic function with a dose more than 200-fold lower than that used in previous studies utilizing systemic delivery. This approach could potentially lead to significant improvements in treatment outcomes for patients suffering from pathological cardiac hypertrophy and other cardiovascular conditions.

## Abbreviations

UTMC: Ultrasound Targeted Microbubble Cavitation
LV: left ventricle
MB: microbubble
