# Supplementary material for: Ultrasound and Microbubble-targeted Delivery of a microRNA Inhibitor to the Heart Suppresses Cardiac Hypertrophy and Preserves Cardiac Function
Source: Theranostics. 2019 Sep 21;9(23):7088–98. doi: 10.7150/thno.34895 (PMC6815962; doi:10.7150/thno.34895)
Supplement: Supplementary file 1 — Supplementary figures and tables. [file thnov09p7088s1.pdf]

# Ultrasound and Microbubble-targeted Delivery of a microRNA Inhibitor to the Heart Suppresses Cardiac Hypertrophy and Preserves Cardiac Function

Jonathan A. Kopechek, Ph.D.,[1,2] Charles F. McTiernan, Ph.D.,[1] Xucai Chen, Ph.D.,[1] Jianhui Zhu, Ph.D.,[1] Maureen Mburu, M.D.,[1] Rafey Feroze, B.Sc.,[1] Daniel A. Whitehurst, B.Sc.,[1] Linda Lavery, B.Sc.,[1] Jissy Cyriac, M.Sc.,[1] Flordeliza S. Villanueva, M.D.[1*]

[1]Center for Ultrasound Molecular Imaging and Therapeutics, Heart and Vascular Institute, Pittsburgh Heart, Lung, Blood and Vascular Medicine Institute, University of Pittsburgh, Pittsburgh, PA, USA

[2]Dept. of Bioengineering, University of Louisville, Louisville, KY, USA

# ONLINE SUPPLEMENTAL MATERIAL

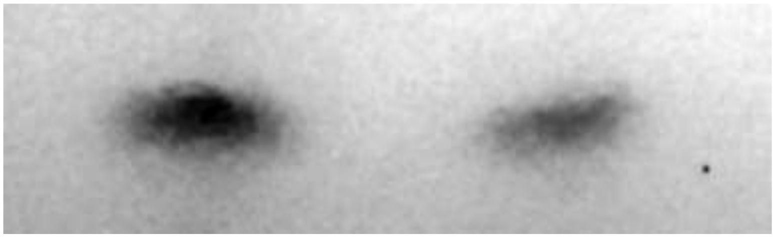


**Figure S1: AntimiR loading on microbubbles.** Methylene blue electrophoresis indicating that 40% of the antimiR added to the formulation could be loaded on to the microbubbles.

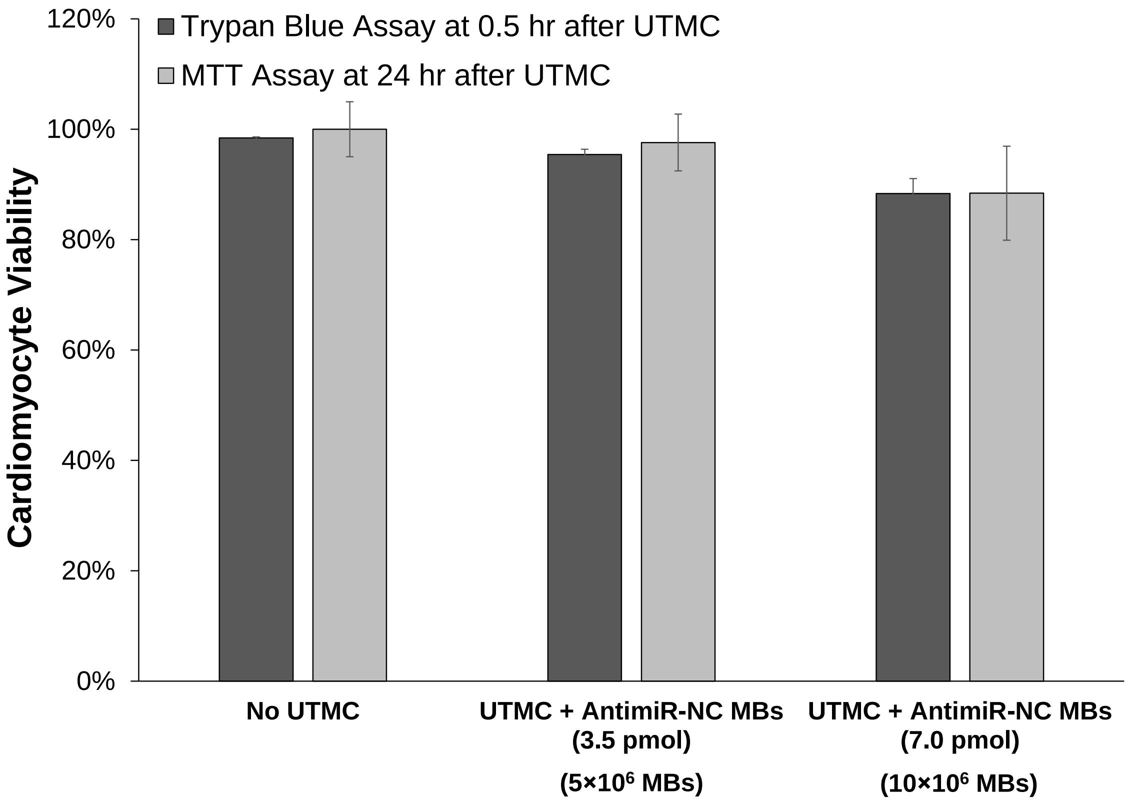


**Figure S2: Viability after ultrasound treatment.** Cardiomyocyte viability following UTMC was not significantly affected by UTMC treatment (*n*=4/group for Trypan Blue Assay, *n*=7-10/group for MTT Assay, *p*=NS).

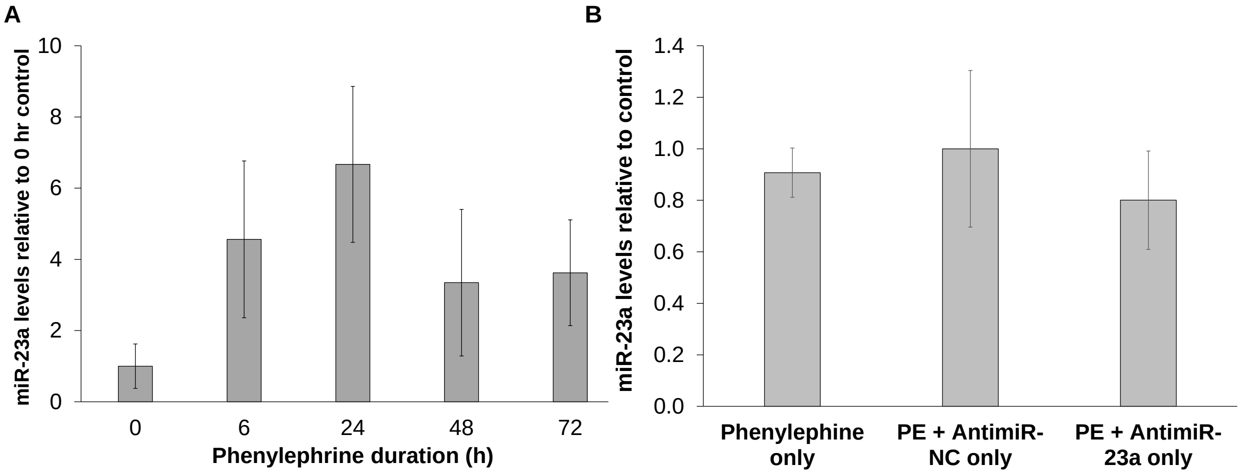


**Figure S3: miR-23a levels in cardiomyocytes.** (A) RT-PCR showing strong trend of increased levels of miR-23a in cardiomyocytes after phenylephrine exposure (*n*=3/group, *p*=0.13). (B) Compared to control groups, no significant difference in miR-23a levels was detected in cardiomyocytes treated with antimiR-23a or antimiR-NC alone (no microbubbles or ultrasound) following 24 h of phenylephrine exposure (*n*=3-6/group). PE = phenylephrine.

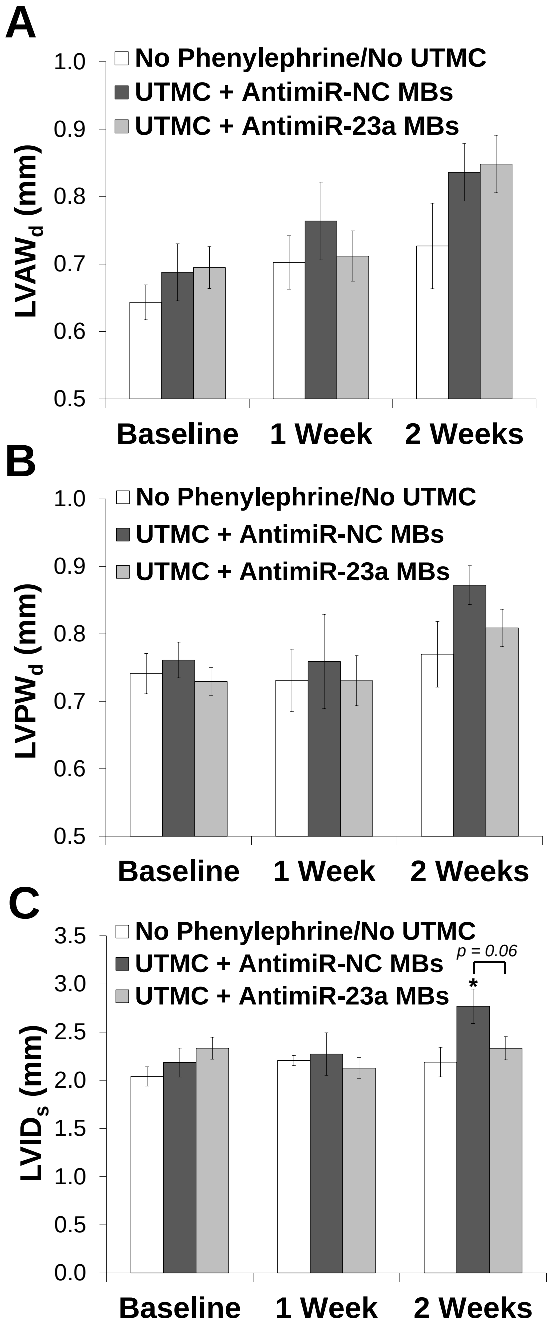


**Figure S4: Diastolic wall thickness and systolic internal diameter.** Echocardiographic analysis of mouse hearts following UTMC targeted delivery of antimiR-23a compared to UTMC delivery of antimiR-NC, or no phenylephrine. (A) Diastolic LV anterior wall thickness, (B) diastolic posterior wall thickness, and (C) systolic LV internal diameter ($n$=5-9 animals/group); UTMC + AntimiR-NC vs UTMC + AntimiR-23a microbubble groups, $p$=0.06; UTMC + AntimiR-NC microbubble group at 2 weeks vs baseline, *$p$=0.03.

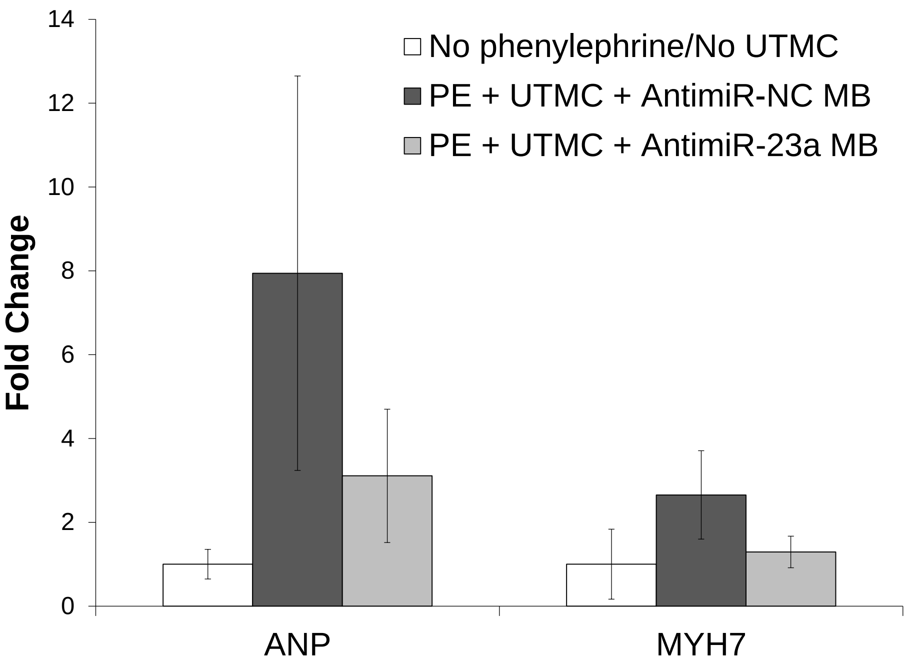


**Figure S5: Expression of hypertrophy markers in mouse hearts.** Myocardial expression levels of hypertrophy markers measured by RT-PCR (*n*=5-9 animals/group).

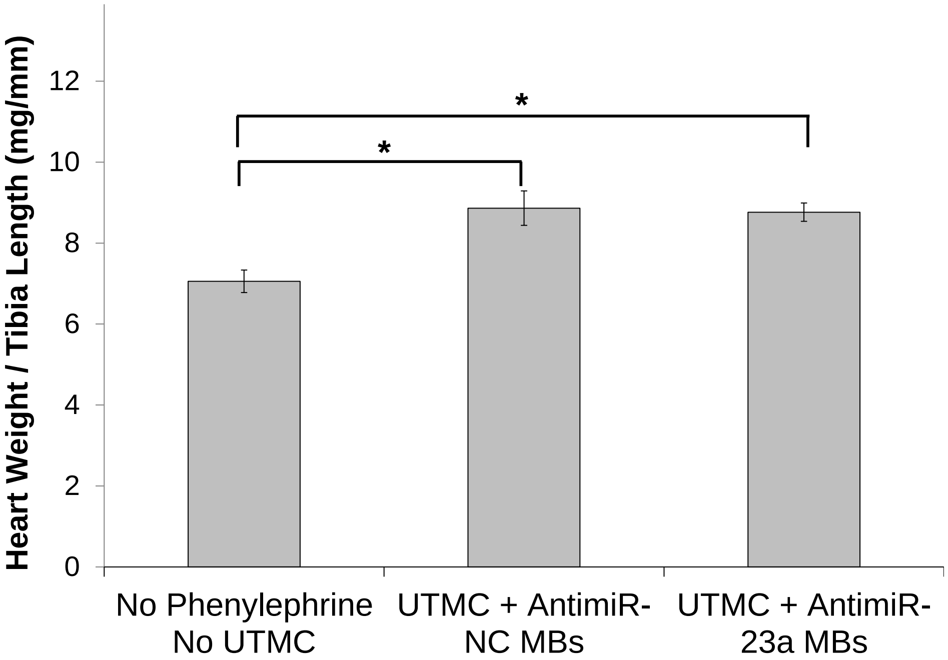


**Figure S6: Heart weights.** Normalized total heart weight measured immediately after sacrifice at 2 weeks (*n*=5-9 animals/group, *$p < 0.05$).
